# Perianal squamous cell carcinoma: A case report

**DOI:** 10.1016/j.ijscr.2021.105739

**Published:** 2021-03-11

**Authors:** Christina Eliachevsky, Erin Templeton, Atul K. Nanda

**Affiliations:** a Medical Student, St. George’s University School of Medicine, Grenada; b Chairman of Surgery, Humboldt Park Health, Chicago, IL, 60622, USA; c Associate Professor of Surgery, St. George’s University School of Medicine, Grenada

**Keywords:** General surgery, Case report, Oncology, Squamous cell carcinoma, Anal margin

## Abstract

**Introduction and importance:**

Perianal carcinomas, though rare, are usually squamous cell carcinoma. Current literature recommends surgical excision for tumors staged T1-T2, N0 without external anal sphincter involvement, however our case demonstrated that tumors with superficial involvement of external sphincter fibers can be resected completely.

**Case presentation:**

A 45-year-old Caucasian male presented with a perianal mass found to be squamous cell carcinoma. Initial imaging suggested the anal sphincter was spared, however intraoperatively tumor cells were found involving superficial external sphincter fibers and a portion was excised to ensure complete removal.

**Clinical discussion:**

Perianal squamous malignancies are often misdiagnosed as more benign conditions. Treatment aims to preserve sphincter function and depends on tumor stage along with anatomical involvement.

**Conclusion:**

Despite superficial muscle infiltration, the T2N0 perianal lesion was curable with surgical resection alone without recurrence or functional deficits reported one year later. This suggests surgical management may be possible in some cases with sphincter involvement.

## Highlights

• Perianal squamous malignancies are often misdiagnosed leading to delay in treatment.
• Surgical management aims to preserve sphincter function.
• Chemoradiation is preferred in cases of suspected sphincter involvement.
• Some superficial muscle fibers may be resected in select cases without loss of function.

## 1 Introduction

Anorectal cancer represents 0.4% of all new cancer diagnoses annually in the US, however in 2017 it only represented 2% of all GI carcinomas [1]. Perianal malignancies accounts for 3–4% within that subset and are five times less common than anal canal neoplasms [2]. While squamous cell carcinoma (SCC) is responsible for most anal margin tumors, they are generally well differentiated and slow growing [3,4]. All of the following work is reported in line with SCARE criteria [5].

Perianal SCC is treated as other cutaneous squamous cell carcinomas [6]. Surgical excision is preferred in stages T1-T2, N0 without involvement of the external anal sphincter [7]. In our patient, preoperative imaging suggested no involvement of the anal sphincter. During excision the tumor was found to extend to the superficial border of the external sphincter and superficial muscle fibers were resected to achieve free margins. The tumor was completely removed without reported incontinence one year later. This suggests surgical excision of perianal SCC with superficial external sphincter involvement may be possible in select cases without functional loss.

## 2 Presentation of case

A 45-year-old married, heterosexual Caucasian male with a history of Asperger’s Syndrome and 60 pack years cigarette use was found to have a perianal lesion during screening colonoscopy due to family history of colon cancer. He came to our clinic for further evaluation where he reported a one-year history of bleeding, pain and foul-smelling clear discharge from the lesion, worsening over the last 3 months. External exam revealed a firm right anterior perianal lesion with everted margins measuring 3 cm without enlarged inguinal lymph nodes. Digital rectal exam was free of masses in the anal canal or rectum.

**Image 1:**
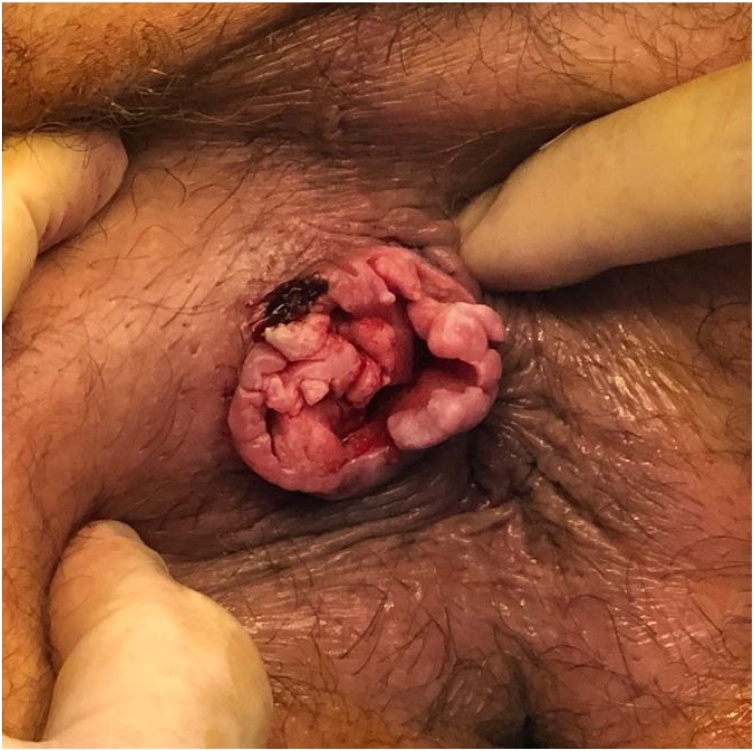
Fungating perianal mass.

Examination with biopsy under anesthesia revealed a mobile, right sided perianal fungating lesion measuring 3.5 × 3.5 cm, and 3 cm deep (Image 1). The medial edge was at the anal verge without involvement of the anus. Clinically the lesion was mobile and didn’t appear to involve the underlying sphincter. Histologically, the biopsy revealed moderately differentiated SCC with verrucous architecture, parakeratosis and a pushing margin, with positive p16 immunoperoxidase staining.

**Image 2:**
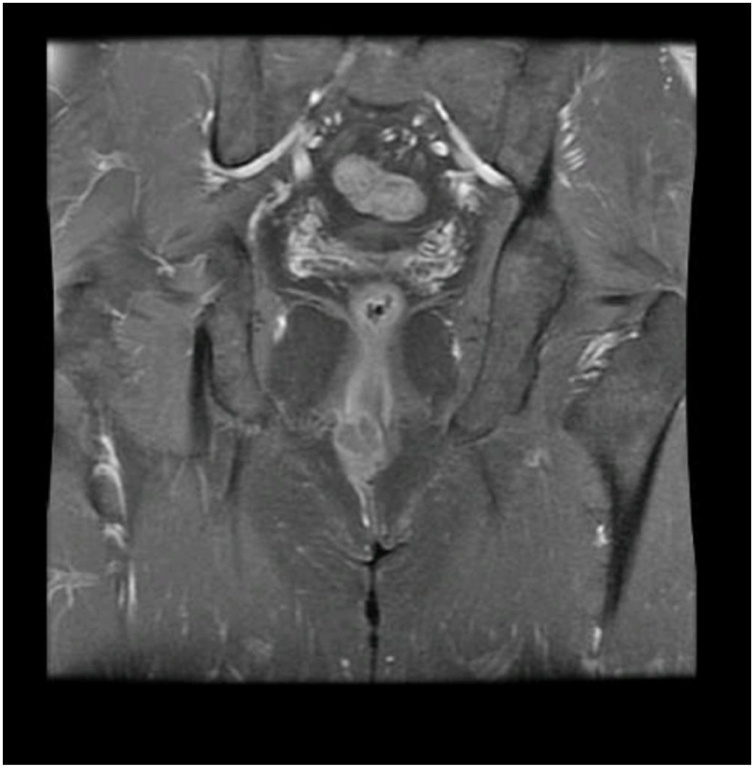
Coronal plane of pelvic MRI showing the mass.

**Image 3:**
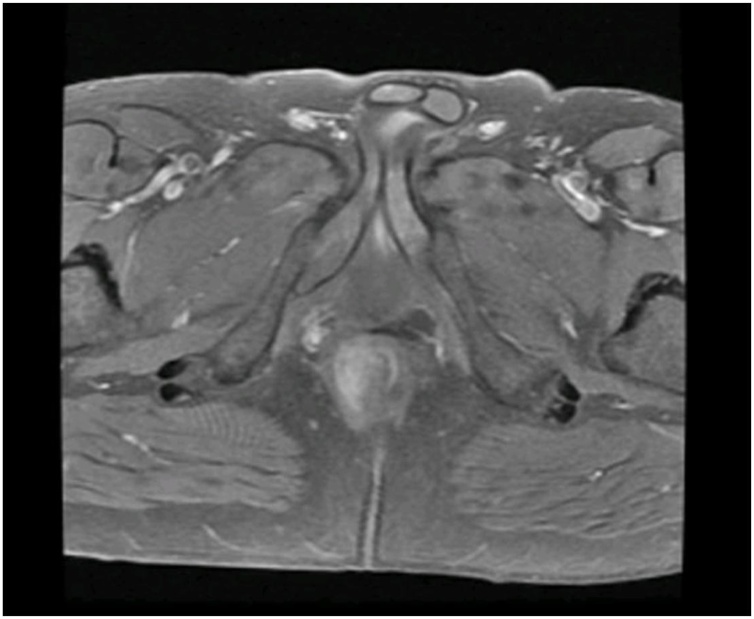
Transverse plane of pelvic MRI depicting the perianal mass.

Subsequent workup was performed. HIV testing was nonreactive. Chest CT showed small 4–6 mm bilateral necrotic pulmonary parenchymal nodules suspicious for metastatic deposits, but PET scan confirmed no evidence of local, regional, or distant metastasis. Pelvic MRI revealed the mass extended to the anal sphincter (Image 2) but did not involve sphincter musculature (Image 3). This suggested stage T2N0M0 disease, and he was planned for local excision with 1 cm margins.

The patient underwent wide local excision of his perianal SCC in a community hospital under the care of an experienced general surgeon. The procedure was performed in the lithotomy position under general anesthesia. A 1 cm circumferential margin was marked around the tumor with the medial aspect extending into the anal canal. Obtaining the medial margin involved resecting a portion of the anal mucosa, and the medial deep margin of the tumor extended to superficial fibers of the external sphincter muscle. Superficial sphincter fibers were resected to obtain negative deep margins. Circumferential 1 cm margins were obtained and ultimately the tumor measured 3.5 cm × 3 cm × 3 cm deep, extending to and involving fibers of the external anal sphincter muscle. The anal mucosa was then approximated, and the anal verge was reconstructed with appropriate approximation at the mucocutaneous junction. The lateral edge was left open and Surgicel was placed to allow drainage and prevent bleeding.

**Image 4:**
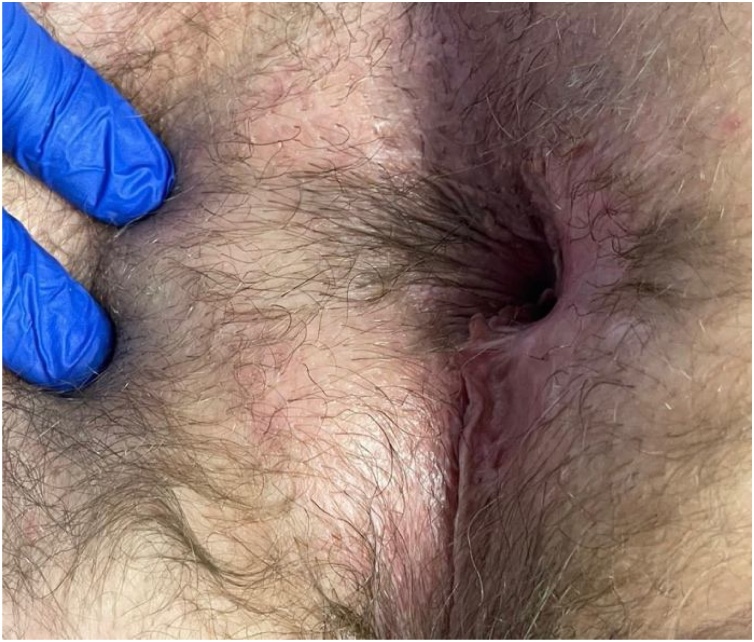
One year after excision.

He was discharged home and instructed to use Miralax 17gm and sitz baths BID to prevent excessive straining and infection. The following day he was seen for Surgicel removal. Ten days later he experienced wound dehiscence from a strenuous bowel movement after he reported stopping Miralax for 36 h. Dehiscence was managed inpatient with local wound care and systemic antibiotics and allowed to heal by secondary intention. Follow up in person was limited due to the COVID-19 pandemic however he was seen via telehealth until he could follow up in person. One year after excision his wound healed completely with scarring (Image 4). Examination and anoscopy revealed no evidence of local recurrence or loss of function to the external anal sphincter or reported fecal incontinence. Avoiding functional deficits remains a goal of treatment and he will return in 3–6 months for DRE and inguinal lymph node palpation [2,19].

## 3 Discussion

While perianal SCC usually presents as an ulcerated lesion with rolled everted edges, it contains histological subtypes including basaloid, transitional, keratinizing, non-keratinizing, verrucous, etc. [6,8]. Current WHO classification no longer distinguishes between variants. Instead they are grouped as well, moderately or poorly differentiated squamous cell carcinomas [8]. This lesion exhibited verrucous architecture with exophytic proliferations of large, pale staining keratinocytes invading the dermis in a pushing pattern. This pushing margin is characteristic of perianal SCC’s preference for local invasion, however larger tumors should raise concern for lymphatic spread [6,8,9]. As in this case, perianal SCC often exhibits positive p16 immunohistochemical staining. P16 is a tumor-suppressor protein surrogate for HPV involvement. While some cases report SCC of the anal margin without positive p16 staining, HPV is widely considered a risk factor [8,[10], [11], [12]]. Other risks include smoking, previous STIs, immunosuppression related to HIV or organ transplant, and hematological or immunological disorders [13]. Experts disagree whether chronic inflammation is a contributing factor [6,14,15]. Common rectal symptoms include bleeding, pain, ulceration, anal discharge, pruritis or a palpable mass, while some patients exhibit enlarged inguinal lymph nodes without perianal symptoms [6,9,11,16]. Treatment is delayed in up to 33% of cases when nonspecific symptoms are misdiagnosed as hemorrhoids, eczema or anal fissures, and biopsy of any perianal lesion not responding to conservative therapy is recommended [2,4,6].

To identify relevant anatomy, the anal margin begins at the anal verge extending along a 5 cm radius along the perianal skin. The anal canal lies superiorly, from the mucocutaneous junction to the beginning of the rectal mucosa [4]. The dentate line lies 1–2 cm above the anal verge and marks the transition of columnar to squamous epithelium at the anal transitional zone (ATZ) [2,6]. The ATZ contains a mixed epithelial lining and is considered important in maintaining fecal continence because it lines the rectum along the areas of the internal and external anal sphincters [2]. Lymphatics of the internal sphincter and anal canal above the dentate line drain to the inferior mesenteric nodes via the submucosal and intramural lymphatics of the rectum, while the anal canal below the dentate line drains via perianal plexuses to external inguinal lymph nodes [2,6]. Lymphatics from the anal margin drain to external inguinal nodes which require examination in suspected perianal malignancy.

Anatomical involvement heavily influences treatment. Well-differentiated perianal lesions up to T2, N0 without suspected sphincter involvement are locally excised with wide margins of 1 cm [7]. Chemoradiation is preferred with advanced tumors, nodal involvement or invasion of the sphincter muscle, while abdominoperineal resection is reserved for large tumors or salvage therapy [2,4,6,17]. Treatment modality aims to preserve sphincter function and avoid a diverting colostomy [2,18]. In this case, we didn’t anticipate having to resect superficial sphincter fibers, however he experienced no functional deficit postoperatively.

While TNM staging provides the most accurate predictor of prognosis, perianal SCC can recur in up to 66% of patients after local resection [1,2,4,9,13]. Posttreatment surveillance involves follow-up in 8–12 weeks with visual inspection and DRE. With complete remission, subsequent follow-up should occur every 3–6 months with DRE and inguinal node palpation for 5 years with anoscopy every 6–12 months and annual CT imaging for 3 years [19].

## 4 Conclusion

Perianal SCC is often delayed in presentation or misdiagnosed as a benign condition. Consider this malignancy in patients with persistent complaints of perianal bleeding, pain, pruritis, or enlarged inguinal lymphatics without known cause. While guidelines recommend resection of moderately to well differentiated cases at T1-T2, N0 without sphincter involvement, our patient demonstrated the tumor can be completely excised with superficial sphincter fibers without functional loss of the musculature.

## Declaration of Competing Interest

None.

## Sources of funding

This research did not receive any specific grant from funding agencies in the public, commercial, or not-for-profit sectors.

## Ethical approval

The study is exempt from ethical approval in our institution.

## Consent

Consent was obtained prior to writing this case report. All other details regarding patient privacy are completed as applicable.

## Author contribution

**Christina Eliachevsky**: Writing – Original Draft, Writing – Review & Editing. **Erin Templeton**: Writing – Review & Editing. **Atul K. Nanda**: Methodology, Writing – Review & Editing, Supervision.

## Registration of research studies

Not applicable.

## Guarantor

Christina Eliachevsky

Erin Templeton

Atul K. Nanda

## Provenance and peer review

Not commissioned, externally peer-reviewed.
